# The Application of Entropy in Motor Imagery Paradigms of Brain–Computer Interfaces

**DOI:** 10.3390/brainsci15020168

**Published:** 2025-02-08

**Authors:** Chengzhen Wu, Bo Yao, Xin Zhang, Ting Li, Jinhai Wang, Jiangbo Pu

**Affiliations:** 1 School of Life Sciences, Tiangong University, Tianjin 300387, China; 2230101323@tiangong.edu.cn; 2 Institute of Biomedical Engineering, Chinese Academy of Medical Sciences and Peking Union Medical College, Tianjin 300192, China; yaobo@bme.pumc.edu.cn (B.Y.); xin_zhang_bme@163.com (X.Z.); liting@bme.cams.cn (T.L.); 3 Tianjin Key Laboratory of Neuromodulation and Neurorepair, Tianjin 300192, China

**Keywords:** information entropy, motor imagery, electroencephalogram, pattern classification

## Abstract

**Background:** In motor imagery brain–computer interface (MI-BCI) research, electroencephalogram (EEG) signals are complex and nonlinear. This complexity and nonlinearity render signal processing and classification challenging when employing traditional linear methods. Information entropy, with its intrinsic nonlinear characteristics, effectively captures the dynamic behavior of EEG signals, thereby addressing the limitations of traditional methods in capturing linear features. However, the multitude of entropy types leads to unclear application scenarios, with a lack of systematic descriptions. **Methods:** This study conducted a review of 63 high-quality research articles focused on the application of entropy in MI-BCI, published between 2019 and 2023. It summarizes the names, functions, and application scopes of 13 commonly used entropy measures. **Results:** The findings indicate that sample entropy (16.3%), Shannon entropy (13%), fuzzy entropy (12%), permutation entropy (9.8%), and approximate entropy (7.6%) are the most frequently utilized entropy features in MI-BCI. The majority of studies employ a single entropy feature (79.7%), with dual entropy (9.4%) and triple entropy (4.7%) being the most prevalent combinations in multiple entropy applications. The incorporation of entropy features can significantly enhance pattern classification accuracy (by 8–10%). Most studies (67%) utilize public datasets for classification verification, while a minority design and conduct experiments (28%), and only 5% combine both methods. **Conclusions:** Future research should delve into the effects of various entropy features on specific problems to clarify their application scenarios. As research methodologies continue to evolve and advance, entropy features are poised to play a significant role in a wide array of fields and contexts.

## 1. Introduction

Brain–Computer Interface (BCI) technology facilitates interaction between the human brain and external devices by recording and decoding neurophysiological signals generated by brain activity. These signals are processed into formats that can be interpreted by external devices, such as computers, enabling communication and control [1]. Common paradigms employed in BCI systems include Steady-State Visual Evoked Potential (SSVEP), P300, and Motor Imagery (MI).

MI is categorized into visual MI and kinesthetic MI. Visual MI involves imagining oneself as an observer watching specific movements from a distance, while kinesthetic MI involves imagining oneself performing specific movements from a first-person perspective [2]. MI signals are bioelectrical signals generated by the brain during the mental simulation of movement, representing an endogenous and spontaneous activity. Different MI tasks induce various brain signals that encompass distinct sensory motor rhythms (SMRs), characterized by increases or decreases in the power of specific frequency bands across different brain regions [3,4]. Compared with P300 and SSVEP, MI only requires the user’s own motor imagery without external stimuli, making the interaction more natural. Furthermore, MI is based on the user’s subjective brain activity, making it more suitable for long-term use and avoiding visual fatigue.

The Common Spatial Pattern (CSP) and its derivatives are among the most prevalent feature-extraction methods employed in MI-BCI data processing [5,6,7,8,9]. CSP extracts feature vectors from SMRs, which are then input into various classifiers for training and classification [10]. However, MI-BCI signals often exhibit significant inter-trial and inter-individual variability, manifesting as complex nonlinear dynamic characteristics. These traditional linear feature-extraction methods struggle to effectively capture the complex characteristics of these signals [11], so the classification of MI-BCI signals is more challenging [12].

Entropy is a measure of the disorder and randomness of a system. In the mid-19th century, Clausius introduced the concept of entropy in the study of the second law of thermodynamics, which is known as thermodynamic entropy [13]. Later, Boltzmann enriched its physical meaning by providing a statistical mechanics interpretation and derived the statistical expression of entropy, known as the Boltzmann relation, which is the entropy in statistical mechanics [13]. Shannon, in 1948, defined a quantity that measures the uncertainty of information and called it information entropy [14]. As research on information entropy deepened, researchers later proposed approximate entropy, sample entropy, permutation entropy, fuzzy entropy, and other types of information entropy. “Information entropy” thus became a general term for this class of computational methods, with Shannon’s originally proposed information entropy being renamed as Shannon entropy. Information entropy, due to its nonlinear characteristics, can effectively describe the complex dynamic behavior of EEG signals and can compensate for the shortcomings of traditional linear feature-extraction methods in MI classification, which is widely used in the feature extraction of physiological signals such as ECG and EEG [14]. The application of information entropy in EEG has mainly focused on the diagnosis of neurological diseases [15], with relatively fewer applications in motor imagery paradigms in brain–computer interfaces. Depending on the type and definition of information entropy, it can be used to measure different aspects of signal characteristics [16]. Using information entropy for feature extraction in motor imagery signals can improve the efficiency of feature extraction and enhance the accuracy of signal classification.

In recent years, although the application of information entropy in MI has increased, related application scenarios remain unclear due to the variety of information entropy types and lack of systematic review. Additionally, comparative studies on different types of entropy in MI (e.g., in neuroscience, motor science, etc.) are relatively scarce, making it difficult to precisely select the most suitable entropy metric in specific scenarios. Therefore, the applicability of entropy in MI remains somewhat limited. This study systematically reviewed articles about entropy applied for MI-BCI classification, summarizing the physical significance and mathematical formulas of commonly used entropy indices in different application scenarios of MI-BCI. This study aimed to help future researchers select the most appropriate entropy to process MI-BCI signals in different scenarios.

## 2. Materials and Methods

This study used keywords such as “MI”, “EEG”, “motor imagery”, “entropy”, and “information entropy” to search for relevant studies in the “Web of Science” and “PubMed” databases from 2019 to 2023. Selection criteria: ① Entropy is used for MI signal-feature extraction; ② Entropy is used to optimize MI classification algorithms; ③ The research topic aligns with the application of entropy in MI; ④ The language is English; ⑤ The paper is a research paper.

Exclusion criteria: ① Duplicate literature; ② Unofficially published papers, such as master’s and doctoral dissertations, conference papers, advertisements, etc.; ③ Unable to obtain the full text of the paper; ④ Review articles.

Our latest search work was performed on 31 December 2023. The titles and abstracts were screened by two independent reviewers. In the second filtering, these full-text articles were then analyzed and evaluated independently by the reviewers. In the case of discrepancies between the two reviewers, a third reviewer decided whether the article should be included. During this process, related studies were progressively included in our final database.

**Figure 1:**
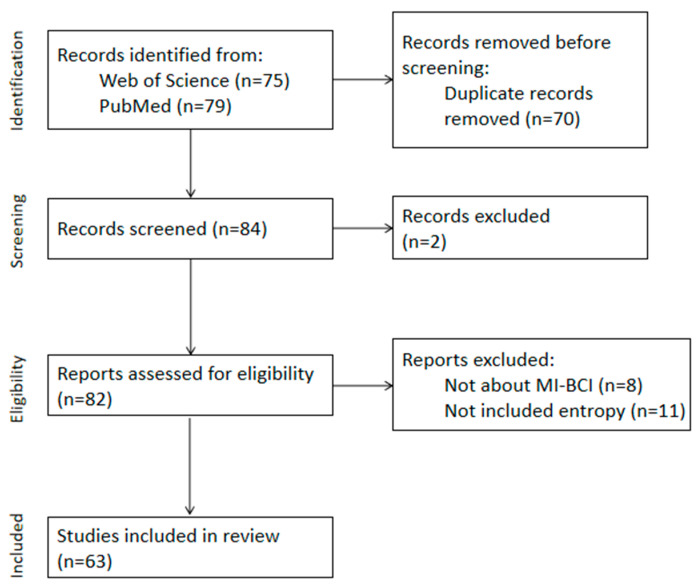
Flow diagram of PRISMA approach used for this systematic review.

Ultimately, 63 studies meeting the criteria from the past five years (2019–2023) were included, with the screening process illustrated in Figure 1. We will list all the information in Appendix B. And we have summarized the journal sources in Appendix C. 

## 3. Result

### 3.1. Physical Significance and Calculation Methods of Entropy

This study summarizes the names, functions, and application ranges of 13 commonly used information entropies in these 63 journal articles (Table 1).

### 3.2. Application of Entropy in MI

After preprocessing the EEG signals of the motor imagery paradigm, feature-extraction algorithms need to be applied to accurately extract key features from the signals. These extracted features are then transformed into feature vectors or feature matrices, which are input into classifiers for systematic training and classification. Finally, classification results are obtained through this process. It is worth noting that the final classification results are often influenced by the choice of feature-extraction methods and the performance of classifiers. Therefore, choosing an appropriate feature-extraction method or improving classifier efficiency can lead to better classification results. This section will introduce the application of entropy in MI from three perspectives: entropy for feature extraction, entropy for optimizing classification algorithms, and multiple entropy fusion classification.

#### 3.2.1. Entropy for Feature Extraction

Entropy, as a nonlinear feature, can be directly used for feature representation of MI signals. Previous research has used seven types of entropy to directly extract features from EEG signals of four different grip forces, combining the obtained entropy values (power spectral entropy, singular spectral entropy, logarithmic energy entropy, sample entropy, fuzzy entropy, permutation entropy, and envelope entropy) into feature vectors and using support vector machines (SVM) for classification [16]. The classification accuracy reached 91.73% in binary classification and 69.59% in four-class classification.

Entropy can also be combined with other feature-extraction methods, and the combination of multiple classification methods can more comprehensively extract signal features, thereby improving classification results. Yang et al. [40] used CSP and improved multiscale permutation entropy fusion methods to extract MI features. Under the same classifier, the average classification accuracy of multi-domain feature extraction increased by 1.52% compared to CSP features alone, and by 32.87% compared to using improved multiscale permutation entropy features alone.

Hou et al. [41] used a new framework based on high-order spectral entropy and CSP to extract features from BCI Competition IV dataset IIa and BCI Competition III dataset IVa, using an SVM algorithm based on RBF kernel function for classification. The highest accuracy on the dataset 2a reached 85%, and the experiment on dataset IVa also achieved good results.

Saha et al. [42] used wavelet-based mean maximum (wMEM), CSP, and regularized common spatial pattern (RCSP) to judge the sensorimotor tasks of the right hand and right foot using the BCI Competition III dataset IVIA and task-specific EEG channels.

Ji et al. [43] proposed a feature-extraction method based on discrete wavelet transform (DWT), empirical mode decomposition (EMD), and approximate entropy. They decomposed the EEG signals into a series of narrowband signals, then used EMD to decompose the sub-band signals, obtaining a set of stationary time series called intrinsic mode functions (IMFs). They then reconstructed the IMFs with appropriate signal selection to obtain the approximate entropy of the reconstructed signal as the corresponding feature vector, and finally used SVM for classification.

Wang et al. [44] mapped EEG signals to feature spaces using time-domain information, statistical measures, wavelet coefficients, mean power, sample entropy, and CSP, using SVM as the intelligent classifier model and sparse logistic regression as the feature selection technique, achieving an accuracy of 79.51%.

Entropy can also participate in channel selection, removing redundant information from EEG signals to improve classification results. Sun et al. [45] calculated the weighted permutation entropy of all sampled electrode channels, selected half of the channels with higher weighted permutation entropy values, then used a binary gravitational search algorithm to search and determine an optimal channel combination. Finally, CSP was used to extract features from the selected channels, and SVM was used to train the classifier. The method achieved accuracies of 88.0% (compared to 57.5% using all channels) and 91.1% (compared to 79.4% using all channels) on two datasets, respectively.

Entropy provides efficient feature-extraction methods, effectively extracting features from different MI signals. Combining entropy with traditional feature-extraction methods can further improve classification accuracy, indicating the necessity of exploring fusion-feature-extraction methods involving entropy. After extracting MI features using entropy or entropy-involved fusion algorithms, most studies employ traditional machine learning classifiers or neural network classifiers for classification.

**Figure 2:**
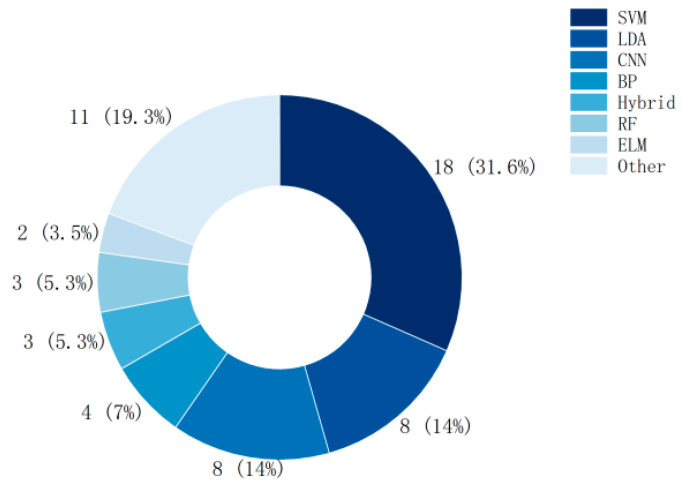
Number of studies using classifiers (BP: Back Propagation Neural Network, RF: Random Forest, ELM: Extreme Learning Machine).

As can be seen from Figure 2, SVM is one of the most widely used classification algorithms, Linear Discriminant Analysis (LDA) and Convolutional Neural Network (CNN) are also frequently used in the field of MI-BCI.

Khan et al. [46] compared the accuracies of different classifiers in the same scenario. The tests were carried out on two datasets, BCI Competition IV dataset 1 and BCI Competition III dataset 4a, using 10-fold cross-validation. On BCI Competition IV dataset 1, the average classification accuracy of the logistic regression classifier reached 90.42%, outperforming other classifiers (such as 89.78% for LDA, 89.20% for Gaussian Naive Bayes, 88.14% for Fine Gaussian SVM, 86.21% for Fine KNN, and 85.57% for Complex Tree). On BCI Competition III dataset 4a, the average classification accuracy of the logistic regression was 95.42%, which was also higher than that of other classifiers (95.06% for LDA, 94.44% for Gaussian Naive Bayes, 92.72% for Fine KNN, 90.66% for Complex Tree, and 87.78% for SVM).

But, there are few direct comparisons of the efficiency differences of different classifiers in processing the same entropy feature-extraction task in current studies, and due to the diversity of data sets adopted by different studies, it has not yet been clearly determined which classifier best matches the specific entropy feature to achieve the best classification effect. Further in-depth research is needed to explore and solve the problem of how to select the most suitable classifier for a specific entropy feature.

#### 3.2.2. Entropy for Optimizing Classification Algorithms

Tang et al. [47] proposed a Conditional Adversarial Domain Adaptation Neural Network (CDAN) that applies densely connected convolutional neurons to obtain high-level discriminative features from the original EEG time series. On this basis, a new cross-entropy-based conditional domain discriminator is introduced to confront the label classifier, learning common subject–invariant EEG features. Alan Preciado-Grijalva et al. [48] explored the application of CDAN and its entropy variant CDAN + E in domain adaptation, reducing the uncertainty between the source domain and target domain through entropy conditioning, thereby improving classification performance. Yu Xie et al. [49] proposed an MI EEG signal classification method based on data augmentation and convolutional neural networks, combined with a cross-entropy loss function, which significantly improved classification accuracy. Rabia Avais Khan et al. [46] proposed a novel framework that enhances MI EEG signal classification accuracy and efficiency through channel selection techniques and entropy-based optimization methods.

Optimizing classification algorithms using entropy is an emerging research direction, with limited research in this area so far. This may be due to the fact that both entropy and neural networks lack good interpretability.

#### 3.2.3. Multiple Entropy Fusion Classification

When using information entropy, multiple entropies can be selected simultaneously, and their results can be fused into a single-feature quantity as a basis for classification. Li et al. [50] used sample entropy, permutation entropy, and CSP to study the impact of massage on decoding EEG rhythms of left/right motor imagery and motor execution (ME) in patients with skeletal muscle pain. Al-Qazzaz et al. [51] combined various entropies in an MI dataset of stroke patients with upper limb hemiparesis. All patients underwent 25 MI-based BCI rehabilitation sessions, followed by assessments to examine EEG neuro-rehabilitation before and after functional changes. Conventional filters and automatic independent component analysis with wavelet transform (AICAWT) techniques were first used for denoising. Then, time, entropy, and frequency-domain attributes were calculated, and effective features were combined into time-entropy-frequency (TEF) attributes. Therefore, the AICAWT and TEF fusion feature set were used to develop an AICA-WT-TEF framework. Then, the SVM, k-nearest neighbor (kNN), and RF classification techniques were tested for MI-based BCI rehabilitation. The study used multiple entropies, namely sample entropy, fuzzy entropy, Tsallis entropy, improved multiscale permutation entropy, multiscale fuzzy entropy, and fine composite multiscale fuzzy entropy.

Khare et al. [52] proposed a simple and flexible MI classification method that uses single-channel adaptive decomposition methods and EEG signal classification methods, employing a multi-cluster unsupervised learning method for channel selection, and flexible variational mode decomposition (F-VMD) for adaptive signal decomposition. The F-VMD decomposition results were feature-extracted using entropy and quartile methods, including approximate entropy, sample entropy, Shannon entropy, logarithmic entropy, permutation entropy, Renyi entropy, and Tsallis entropy. These features were then classified using a flexible extreme learning machine (F-ELM).

Wu et al. [53] used multivariate empirical mode decomposition (MEMD) to decompose EEG signals into multiple intrinsic mode functions (IMFs). CSP was applied to highly correlated IMF functions to extract AR coefficients and energy entropy, fuzzy entropy, and multiscale entropy results as classification features. After optimization using genetic algorithms, classification was performed.

Chen et al. [54] extracted four types of entropy from the collected EEG signals, including Shannon entropy amplitude measurement, Shannon entropy phase synchronization measurement, wavelet entropy, and sample entropy. Principal component analysis was then used for dimensionality reduction, and SVM was used for classification. The method was evaluated using the BCI Competition 2003 Dataset III. The experimental results showed that the classification results of using Shannon entropy amplitude measurement, Shannon entropy phase synchronization measurement, wavelet entropy, and sample entropy alone were 81.05%, 78.91%, 80.97%, and 81.52%, respectively. The fusion method based on these four entropies achieved better classification performance, with an accuracy of approximately 88.36%. The results showed that multiple entropy fusion classification results in higher accuracy compared to single entropy.

Different entropies have unique calculation methods and intrinsic meanings. The comprehensive application of multiple entropy features allows for a multidimensional characterization of signals [51], thereby improving classification efficiency.

**Figure 3:**
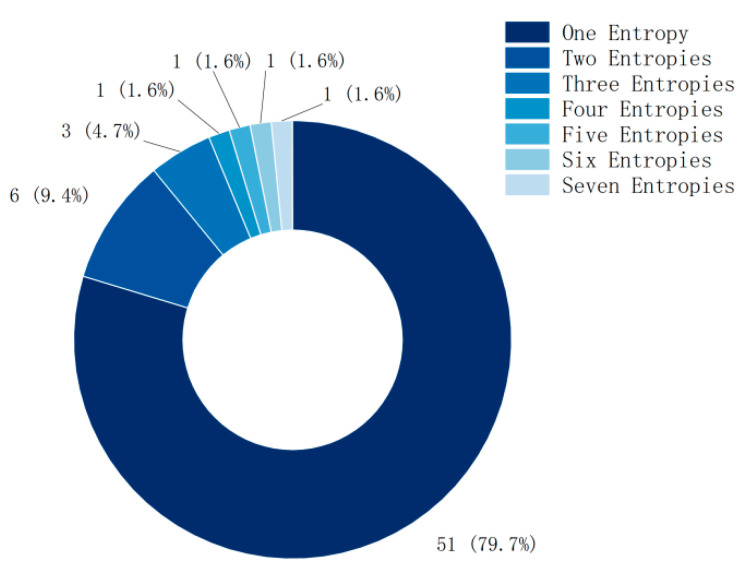
The number of entropies used in each study.

Although the method of combining multiple entropies has achieved better feature- extraction performance in MI classification, nearly 80% of the current research still focuses on a single entropy (as shown in Figure 3), and the research using multiple entropies also focuses on two or three entropies.

Subsequent research should use more entropies for feature extraction and classification of MI.

### 3.3. Number of Research of Entropy Features in MI-BCI

**Figure 4:**
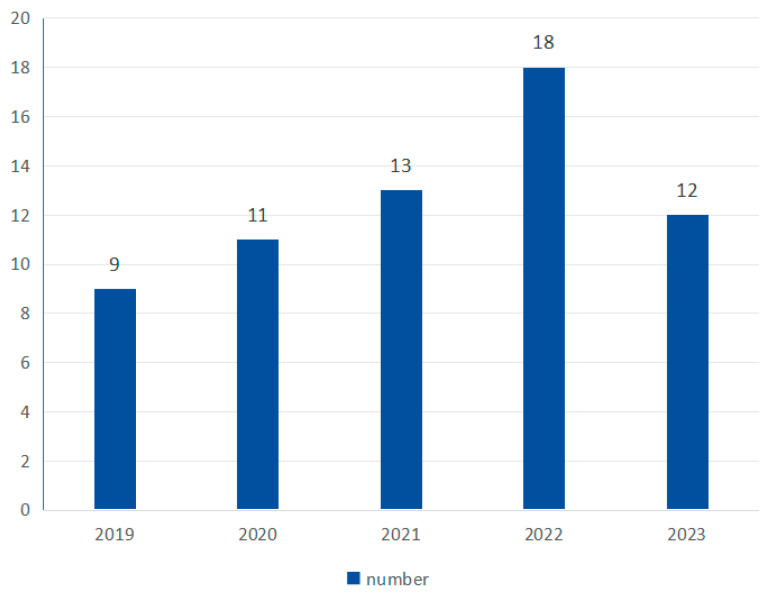
Yearly research number.

As shown in Figure 4, the number of studies shows an overall upward trend, with 9 in 2019, 11 in 2020, 13 in 2021, 18 in 2022, and 12 in 2023.

### 3.4. Accuracy of Entropy Features in MI-BCI

**Figure 5:**
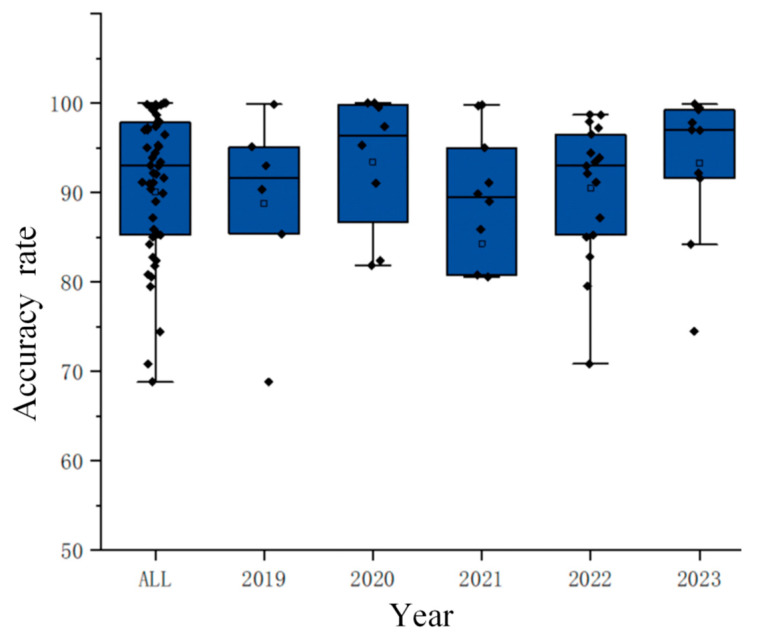
Yearly accuracy distribution chart. (The diamond represents the accuracy of each study, and the hollow square represents the average value of the data in this group).

As shown in Figure 5, most studies have achieved accuracy rates above 80%, with more than half reaching accuracy rates of over 90%, and some studies even achieving peak accuracy rates exceeding 99%. This fully demonstrates that entropy has high classification efficiency in motor imagery paradigms.

### 3.5. Entropy Type in MI-BCI

**Figure 6:**
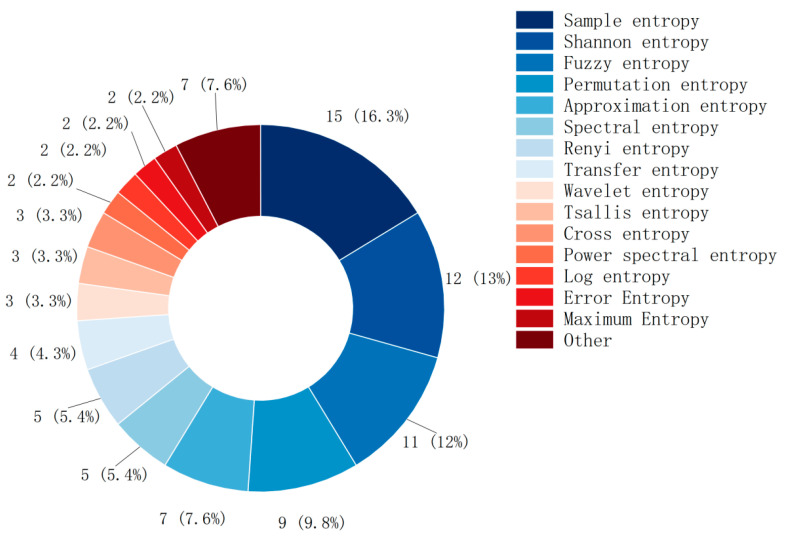
Types of entropy and their usage.

This study conducted a statistical analysis of the most commonly used entropy fea-tures in the literature. Most MI-BCI studies use simple and well-defined entropy features, as shown in Figure 6. The usage frequency in descending order is: sample entropy (15), Shannon entropy (12), fuzzy entropy (10), permutation entropy (8), approximate entropy (7), spectral entropy (5), and Renyi entropy (5).

### 3.6. Selection of Datasets

**Figure 7:**
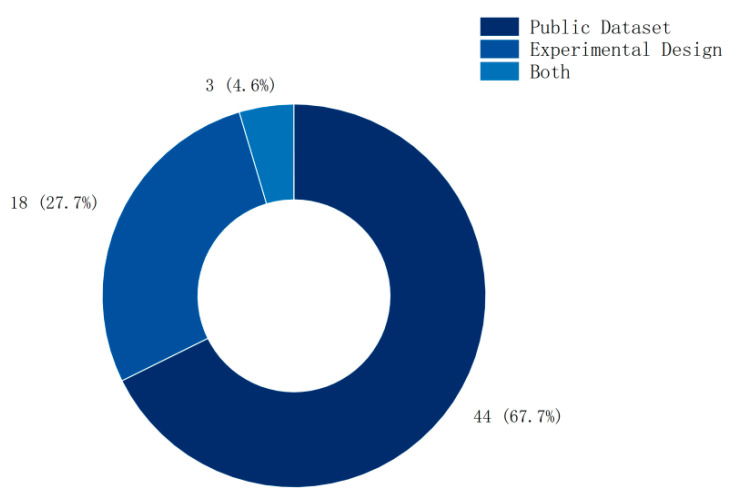
Dataset distribution.

As shown in Figure 7, among the methods for validating entropy features, public dataset validation accounts for 67%, experimental design accounts for 28%, and only 5% combine both. 

## 4. Discussion

Entropy-based feature-extraction and classification methods have been widely used in the field of physiological signal feature extraction due to their simplicity and efficiency. Applying entropy to motor imagery signal classification is an emerging method with broad application prospects. As shown in Figure 4, in recent years (2019–2023), this research direction has gradually become very popular.

### 4.1. Comparison with Other Methods

Currently, the most widely used feature-extraction method for MI-BCI data is the common spatial pattern (CSP) and its derivative methods [5,6,7,8,9]. Since the CSP algorithm adopts a strict linear-mode assumption relationship in the acquisition of source signals and the recording of electroencephalogram (EEG) signals, it is overly restricted by the original recording parameters of the subjects, such as the signal time period, filtering frequency band, number of electrode channels, etc. EEG signals are non-stationary and non-linear, so the CSP algorithm cannot accurately and effectively describe the characteristic signals of the brain. As a non-linear measurement method, entropy is more friendly to non-linear signals.

CSP mainly conducts feature extraction based on spatial filters. Therefore, for movements with significantly different movement areas, CSP can quantify feature quantities with large differences. However, for fine movements with similar movement areas, it is difficult to distinguish them using CSP. Entropy is not affected by spatial position.

The CSP algorithm can only consider the EEG signals of two categories. Thus, when dealing with multi-classification problems, one vs. rest or one vs. one methods need to be used to perform CSP for feature extraction multiple times. In multi-classification, entropy can extract the entropy values of multiple states at once, reducing the calculation steps.

In terms of classification accuracy, Li et al. [55] use fuzzy entropy to extract MI information to construct feature vectors, and use SVM classifier for classification, the final result of which (99.43%) is higher than the traditional method (CSP: 86.67%, FBCSP: 90.50%, DFBCSP: 91.40%, SFBCSP: 92.05%, SWCSP: 93.0%).Yang et al. [40] found that for the same subject and the same classifier, the average classification accuracy of multi-domain feature extraction increased by 1.52% compared to the CSP feature results, and the average classification accuracy increased by 32.87% compared to the IMPE feature classification results. Tang et al. [47] found that, on the High Gamma Dataset, the accuracy of the entropy method (95.3%) was higher than that of FB-CSP (91.2%). Lv et al. [56] found that, compared with the common spatial pattern (CSP) algorithm, the entropy method has significant advantages.

This shows that in some scenarios, compared with CSP, entropy has the characteristics of simple calculation and high accuracy.

### 4.2. Frequently Used Entropy Features in MI-BCI

Compared to other entropy metrics, sample entropy is less sensitive to noise and changes in data length, making it more reliable and stable when processing real EEG signals. In MI-BCI experiments, EEG signals are often disturbed by various noises, and the anti-interference ability of sample entropy makes it an ideal tool for analyzing these signals, which is why it is frequently used. Shannon entropy is a basic concept in information theory used to quantify uncertainty or the amount of information. Its intuitive definition and relatively simple calculation make it easily accepted and applied by researchers in MI-BCI studies. Similar to sample entropy, fuzzy entropy is also suitable for analyzing nonlinear signals. In MI-BCI, this capability allows fuzzy entropy to more accurately capture the nonlinear characteristics of brain activity. The frequent use of sample entropy, Shannon entropy, and fuzzy entropy in MI-BCI literature is mainly due to their advantages in processing complex and nonlinear signals, anti-interference capability, information theory foundation, intuitive understanding, broad application scenarios, fuzziness and uncertainty, and parameter flexibility.

### 4.3. Single- or Multiple-Entropy Features in MI-BCI

The combination of multiple entropies in MI classification indeed achieves better feature-extraction performance, reflecting the advantages of different entropy metrics in capturing different aspects of EEG signals. However, in the current literature review, most studies (79.7%) still focus on single entropy, possibly because single entropy is relatively simple to calculate and intuitive, and, in some cases, already meets the research needs. Nevertheless, a portion of the studies (about 14.1%, i.e., 9.4% using two entropies and 4.7% using three entropies) explored methods of combining multiple entropies and found that this approach significantly improves classification accuracy in different paradigms (8–10%).

Multiple-entropy features have certain advantages in fusion, as different entropy metrics (e.g., sample entropy, Shannon entropy, fuzzy entropy) focus on different aspects of EEG signal complexity, information content, fuzziness, etc. Their combination allows for a more comprehensive extraction of EEG signal features, providing good complementarity. Additionally, the combination of multiple entropies can offer richer feature information, helping to improve classifier performance and thereby enhance classification accuracy. Particularly in complex MI-BCI tasks, the combination of multiple entropies can more accurately reflect the state of brain activity. Furthermore, different MI task paradigms may require different feature-extraction methods. The combination of multiple entropies can be flexibly adjusted to suit the needs of different paradigms, achieving high classification accuracy in various paradigms.

Different entropies have different calculation methods, and using multiple entropies will increase the computational cost and time more than using a single entropy. The single entropy calculation is relatively simple, easy to implement and deploy. In resource-limited or time-sensitive situations, researchers are more inclined to choose single entropy for analysis. Moreover, single entropy has more research basis than multiple entropy. Although the combination of multiple entropies is gaining attention with the deepening of research, single entropy is still the main choice.

To further improve the performance and application effectiveness of MI-BCI systems, actively exploring the combination of multiple entropies may be a future research direction. With the continuous development of computing technology and the increasing availability of computing resources, the combination of multiple entropies will also become easier to implement and deploy.

### 4.4. Application Scenarios of Entropy Features in MI-BCI

Most of the research remains focused on the study of feature-extraction and classification methods, with only a few studies conducted in real-world environments. Choy et al. [35] applied entropy in the motor imagery of stroke patients in order to explore whether motor imagery and virtual reality can help activate the motor cortex of stroke patients. Li et al. [57] used entropy to explore the relationship between motor imagery and age-related fatigue in the CNN classification of EEG data. Al-Qazzaz et al. [51] used entropy to explore a motor-imagery-based rehabilitation assessment scheme for stroke patients.

The dominance of public dataset validation in current research reflects researchers’ pursuit of reproducibility and convenience. However, this does not mean that these studies are diverse, as most studies focus on a small subset of publicly available datasets, or use only one publicly available dataset. However, to clarify the application scenarios of entropy features more precisely, researchers need to design experiments of different types and simultaneously compare the classification results of public datasets to explore the application scenarios of different entropies. With the continuous innovation and improvement of research methods, entropy features will play an important role in disease diagnosis and rehabilitation.

## 5. Conclusions

For subsequent researchers to use entropy features, they can prioritize the use of multiple common entropies (Sample entropy, Shannon entropy, Fuzzy entropy, Permutation entropy, Approximate entropy, Spectral entropy, and Renyi entropy) in combination, and use multiple common classifiers (SVM, LDA, CNN, BP Neural Network, RF, ELM) to classify simultaneously in order to find the best classification results.

Studies have been carried out to compare the feature-extraction and classification performance of different entropies on the same research object in order to explore the best matching entropy of different research objects. Based on this, it is expected to establish a motor imagery paradigm brain–computer interface feature-extraction and classification scheme that integrates multiple feature-extraction methods and adopts multiple classifiers for weighted classification. At the same time, the exploration of the applicable scope of different entropy features needs to continue, aiming to form a consensus on entropy features in motor imagery.

## 6. Limitation

This study has the following limitations due to the focus and inherent shortcomings of the articles in the areas reviewed.

In terms of application scenarios, we did not explore the sociodemographic characteristics (gender, age, type of neurological pathology) of individuals with EEG, the time of EEG recording, or the number of EEG electrodes used. This is because most of the retrieved studies focused on data processing and did not specify these details.

In terms of computing entropy, we did not explore the computational cost of different entropies and the cost of implementing multi-entropy, because this is difficult to compare due to the varying amounts of data and computing equipment used in different studies. We also did not explore the impact of entropy parameters on classification performance, because different studies used different datasets and classifiers, and comparing them without controlling for variables is unscientific.

In terms of classifier selection, we did not explore how to improve the interpretability of entropy and neural networks.

We hope that future studies will focus on these points and improve upon the research in this field.

## Figures and Tables

**Table 1:** The name, function, and scope of application of information entropy appearing in 63 research papers.

Entropy	Function and Scope of Application	References
Shannon Entropy	The numerical value defined by Shannon to quantify information uncertainty or measure system complexity can be used to quantify EEG signals.	[17]
Power Spectral Entropy	It reflects the distribution of the power spectrum and belongs to the information entropy of the frequency domain. It is applicable to signals with certain frequency differences. The higher the power spectrum entropy value, the more complex and chaotic the signal is.	[18,19,20,21]
Singular Spectral Entropy	The complexity of the data is measured by analyzing the variance distribution of the eigenvectors in the singular value decomposition; higher entropy values indicate a more uniform and complex distribution.	[22]
Log Energy Entropy	It is able to describe the complexity of EEG sub-bands and help successfully classify EEG data by quantifying the variability of signal energy distribution in different frequency bands.	[23,24]
Approximate Entropy	Used to quantify the regularity of EEG time series, and is more suitable for signals with more random components. In general, approximate entropy measures correlation, persistence and regularity of data, that is, lower approximate entropy values indicate that a series is repetitive and predictable, and thus higher approximate entropy values indicate more uncertainty.	[15,25]
Sample Entropy	As an improved form of approximate entropy, it is used to evaluate the complexity of EEG signals. It reduces the deviation of approximate entropy and avoids the influence of signal length.	[26]
Fuzzy Entropy	It is a method for calculating sequence complexity proposed from the sample entropy theory. Fuzzy entropy uses an exponential function to fuzzify the similarity formula, overcoming the defects of sample entropy being sensitive to data length and discontinuous boundaries. Due to the monotonicity and continuity of the fuzzy exponential function, the fuzzy entropy changes smoothly and continuously with the change of parameters. This allows more detailed marginal information to be obtained.	[27,28,29,30]
Permutation Entropy	Assess the complexity of time series by comparing neighboring values, mapping to ordered patterns, identifying nonlinear signals, reducing problem space, and increasing robustness to noise.	[20,27]
Transfer Entropy	A model-free metric based on information theory can be used to calculate the amount of directed information transfer between two systems as a rigorous derivation of a Wiener causal measure.	[31]
Renyi Entropy	It is a generalization of Shannon entropy and represents the spectral complexity of a signal.	[32,33,34]
Spectral Entropy	The regularity of the time series corresponding to the uniformity of the power spectrum distribution is calculated using Shannon entropy and the power spectrum of the signal.	[35]
Wavelet Entropy	As a combination of wavelet decomposition and entropy, it aims to evaluate the chaos intensity of EEG signals.	[36]
Tsallis Entropy	It is a generalized entropy that describes the uncertainty or disorder of the microscopic state within the system, and can be used to deduce the importance of features in the EEG signal. The more uncertain the microscopic state of the system is, the larger the value of Tsallis entropy, indicating that the disorder or complexity of the system is higher.	[37,38,39]

Please refer to Appendix A for commonly used entropy calculation methods.

## Appendix A. Entropy Calculation Method

1. Shannon entropy [17]

$$H_{\mathrm{sh}}=-\sum_{n=1}^{x}P_{n}\log_{2}P_{n}$$

where $P_{n}$ is the nth element of the feature, and x is the total number of features.

2. Power spectrum entropy [18,19,20,21]

$$PSE_{i}=-\sum_{i=1}^{n}PSD_{i}InPSD_{i}$$

where PSD is the power spectral density

$$PSD(\omega_{i})=\frac{1}{N}|X(\omega_{i}){|}^{2}$$

3. Singular spectrum entropy [22]

$$SVDE_{n}=-\sum_{i=1}^{1}\frac{\lambda_{i}}{\sum \lambda }In\frac{\lambda_{i}}{\sum \lambda }$$

where $\lambda_{i}$ is the singular value of the diagonal matrix, and ∑λ are the traces of the diagonal matrix used to normalize the singular value.

4. Log energy entropy [23,24]

$$LogEn=\sum_{i=1}^{N}\log(y_{i}^{2})$$

where N is the length of the time series signal and $y_{i}$ is the ith sample of the signal.

5. Approximate entropy [15,25]

$$AppxEn=\Theta^{m}-\Theta^{m+1}$$

$$\Theta^{m}=\frac{1}{N-m+1}\sum_{i=1}^{N-m+1}\log(\varepsilon_{i}^{m})$$

where the empirical dimension parameter m = 2 and the threshold ε = 0.15.

6. Sample entropy [26]

$$SampEn=-In\frac{P_{a}}{P_{b}}$$

where, $P_{a}$ and $P_{b}$ are the conditional probabilities of occurrence of similar patterns in the sequences a and b of the signal, respectively.

7. fuzzy entropy [27,28,29,30]

$$Fu\mathrm{En}(m,r)=\underset{x\to \infty }{\lim}[In\phi^{m}(r)-In\phi^{m+1}(r)]$$

where, $\phi^{m}(r)=\frac{1}{N-m}\sum_{i=1}^{N-m}\left(\frac{1}{N-m-1}\sum_{j=1j\neq i}^{N-m}\mu (d_{ij}^{m+1},r)\right)$

8. Permutation entropy [20,27]

$$PermEn=-\sum_{i}^{D!}\rho_{\pi_{i}}In\rho_{\pi_{i}}$$

9. Transfer entropy [31]

$$\mathrm{TE}\left(X\to Y\right)=\sum_{y_{t+1},y_{t}^{n},x_{t}^{m}}p\left(y_{t+1},y_{t}^{n},x_{t}^{m}\right)\log\frac{p\left(y_{t+1}|y_{t}^{n},x_{t}^{m}\right)}{p\left(y_{t+1}|y_{t}^{n}\right)}$$

where, $X=x_{t}$ and $Y=y_{t}$ are two time series that can be approximated by Markov processes. $x_{t}^{m}=\left(x_{t},\ldots ,x_{t-m+1}\right)$, $y_{t}^{n}=\left(y_{t},\ldots ,y_{t-n+1}\right)$, m and n are the orders of X and Y in the Markov process respectively.

10. Renyi entropy [32,33,34]

$$H_{\mathrm{re}}=\frac{1}{1-\beta }\ln\left(\sum_{n=1}^{x}E_{n}^{\beta }\right)$$

where $E_{n}$ is the probability of the system, β is the order (β≠1), and x is the number of phases.

11. Spectral entropy [35]

$$H_{\mathrm{spec}}=-\sum_{f_{0}}^{f_{n}}\hat{p}\left(f\right)\log_{2}\left(\hat{p}\left(f\right)\right)$$

where,

$$\hat{p}\left(f\right)=\frac{p\left(f\right)}{\sum_{f_{0}}^{f_{n}}p\left(f\right)}$$

where, $p\left(f\right)$ is the power spectral density; $\hat{p}\left(f\right)$ is the normalized power spectral density; $f_{0}$ and $f_{n}$ are the first and last frequencies in the integral frequency range, respectively.

12. Wavelet entropy [36]

$$H_{W}=\sum_{p<0}H_{p}\ln\left(H_{p}\right)$$

where, $H_{p}$ represents the probability distribution of the EEG signal, and P represents different resolution levels.

13. Tsallis entropy [37,38,39]

$$H_{\mathrm{tsallis}}=\frac{1}{1-\beta }\left(1-\sum_{n=1}^{x}E_{n}^{\beta }\right)$$

where n is the number of features, E is the probability distribution, and β is a real parameter (β≠1), expressed as the entropy index.

## Appendix B. Research Statistical Table

**Table:**

Years	Authors	Entropy Type	Classifier Type	Dataset *	Accuracy
2023	[58]	Differential entropy	GBDT	0	96.96
2023	[59]	Wavelet entropy	RF	0	99.26
2023	[60]	Spectral entropy	SVM	0	84.19
2023	[61]	Shannon entropy	DRN	0	91.6
2023	[62]	Wavelet entropy	SVM	0	97
2023	[63]	Shannon entropy, Renyi entropy, Sample entropy, Permutation entropy, Approximate entropy	NB, SVM, RF, KNN	0	97.83
2023	[30]	Fuzzy entropy	LDA	0	92.14
2023	[35]	Spectral entropy	LDA	1	/
2023	[64]	Renyi entropy, Shannon entropy	LDA	0	99.87
2023	[17]	Shannon entropy	ELM	0	99.48
2023	[65]	Tsallis Entropy, Dispersion Entropy, Shannon entropy	RF, KNN	0	74.48
2023	[49]	Cross entropy	CNN	0	97.61
2022	[66]	Transfer entropy	CNN-LSTM	1	97.21
2022	[40]	Permutation entropy	Decision Tree, LDA, Naive Bayes, SVM, KNN, ECPA	1	91.15
2022	[67]	Sample entropy	BP network	0	93
2022	[19]	Power spectral entropy	SVM	0	85.24
2022	[44]	Sample entropy	SVM	1	79.51
2022	[68]	Approximate entropy	XGBO	0	94.44
2022	[69]	Spectral entropy	LDA	0	98.67
2022	[70]	Spectral entropy	Decision Tree	0	98.71
2022	[56]	Approximate entropy, Sample entropy, Fuzzy entropy, Multiscale entropy	SVM	0	97.88
2022	[71]	Sample entropy	SVM	1	93.89
2022	[57]	Rhythm entropy	CNNs	1	82.81
2022	[72]	Transfer entropy	LDA	1	92.1
2022	[73]	Shannon entropy, Correlation entropy, Conditional entropy	Ego-CNNs	0	93.4
2022	[74]	Rayleigh entropy	SLDA	0	96.5
2022	[41]	Shannon entropy	SVM	0	85
2022	[75]	Information entropy	MSTL-MCF	0	70.86
2022	[76]	Shannon entropy	3-D-CNN	0	87.15
2022	[46]	Shannon entropy	LRC	0	95.42
2021	[77]	Fuzzy entropy	SVM	1	80.56
2021	[78]	Sample entropy	/	1	/
2021	[79]	Sample entropy	SNN	1	89.9
2021	[45]	Permutation entropy, Weighted permutation entropy	SVM	0	91.1
2021	[80]	Renyi entropy	SVM	0	31.14
2021	[81]	Sample entropy	/	1	/
2021	[50]	Sample entropy, Permutation entropy	BiLSTM	1	89
2021	[82]	Sample entropy, Permutation entropy	CNN	0	/
2021	[83]	Shannon entropy	LDA	0	99.8
2021	[84]	Cross entropy	CNN	0	80.8
2021	[85]	Multiscale entropy	FLDA	2	85.89
2021	[86]	Renyi entropy	TE-base	0	95
2021	[51]	Sample entropy, Fuzzy entropy, Tsallis entropy, Improved Multiscale Permutation entropy, Multiscale Fuzzy Entropy, Refined Composite Multiscale Fuzzy entropy	RF	1	99.66
2020	[87]	Maximum entropy	SVM	0	81.83
2020	[88]	Sample entropy	ELM	1	91
2020	[89]	Approximate entropy, Fuzzy entropy	/	0	97.4
2020	[90]	Transfer entropy	/	2	/
2020	[47]	Cross entropy	CDAN	0	95.3
2020	[91]	Sample entropy	SVM	1	99.5
2020	[92]	Multiscale Fuzzy entropy	BP	0	100
2020	[93]	Spectral entropy	/	1	/
2020	[52]	Approximate entropy, Sample entropy, Shannon entropy, Logarithmic entropy, Permutation entropy, Renyi entropy, Tsallis entropy	FFMIC	0	100
2020	[94]	Cross entropy	FBSF-TSCNN	0	82.4
2020	[47]	Shannon entropy	CDAN	0	94.3
2019	[53]	Energy entropy, Fuzzy entropy, Multiscale entropy	KNN	0	85.36
2019	[42]	Wavelet-based average maximum entropy	/	0	90.36
2019	[95]	Shannon wavelet entropy, Logarithmic entropy	LS-SVM	0	93
2019	[55]	Improved refined composite multivariate multiscale fuzzy entropy	SVM	0	99.86
2019	[43]	Approximate entropy	SVM	0	95.1
2019	[96]	Approximate entropy	/	1	/
2019	[97]	Symbolic transfer entropy	LDA	0	68.8
2019	[98]	Minimum error entropy	/	0	/
2019	[48]	Shannon entropy	CDAN	0	Under 70

*: In the dataset column, 0 represents public datasets, 1 represents private datasets, and 2 represents both.

## Appendix C. Journals Used in This Review

**Table:**

Journals	Number
Entropy	8
Frontiers in Neuroscience	6
BIOMEDICAL SIGNAL PROCESSING AND CONTROL	4
IEEE Transactions on Neural Systems and Rehabilitation Engineering	3
Asian Journal of Control	2
BioMedical Engineering OnLine	2
Electronics	2
IEEE Journal of Translational Engineering in Health and Medicine	2
Journal of Neuroscience Methods	2
Sensors	2
Applied Sciences	1
arXiv	1
Behavioral Brain Research	1
Biocybernetics and Biomedical Engineering	1
Biosensors	1
Brain Sciences	1
Cognitive Neurodynamics	1
Computational Intelligence and Neuroscience	1
Computer Methods in Biomechanics and Biomedical Engineering	1
Computers in Biology and Medicine	1
Evolving Systems	1
Expert Systems with Applications	1
Frontiers in Aging Neuroscience	1
Frontiers in Human Neuroscience	1
Frontiers in Neuroinformatics	1
IEEE Access	1
IEEE Sensors Journal	1
IEEE Signal Processing Letters	1
IEEE Transactions on Computational Social Systems	1
IEEE Transactions on Neural Networks and Learning Systems	1
International Journal of Pattern Recognition and Artificial Intelligence	1
Journal of Neural Engineering	1
Mathematical Biosciences and Engineering	1
Multimedia Tools and Applications	1
Neuroscience Letters	1
Neuroscience Research	1
PLOS ONE	1
Power and Entropy	1
Signal	1
Wireless Communications and Mobile Computing	1
